# News and Coming Events

**Published:** 1909-08-21

**Authors:** 


### News and Coming Events.

Under the presidency of the United States Ambassador, Professor Osler will deliver the inaugural address of the winter session of the London School of Tropical Medicine on Tuesday, October 26.

The office of the Secretary-General of the International Congress in Buda Pest has been removed to viii Muzeum-Korüt, 6-8, Buda Pest, where all communications relative to the Congress should be addressed.

Dr. W. Bevan Lewis, Medical Superintendent and Director of the West Riding Asylum, Professor of Mental Diseases in Leeds University, has been elected President of the Medico-Psychological Association of Great Britain and Ireland.

According to the *Times* the Local Government Board is in sympathy with the views which have been strongly expressed, and which were recently set forth in a petition to the Croydon County Council, against the scheme for the demolition of the Whitgift Hospital for the purpose of the North-end widening scheme.

The death is announced of Dr. Theodore Davis, M.D., F.R.C.S., of Walton-in-Gordano, Clevedon, Somerset. Dr. Davis, who was a student of Queen's College, Birmingham, and St. Bartholomew's Hospital, was seventy-five years of age, and retired from practice some years ago.

Dr. Breinl, of the Liverpool School of Tropical Medicine, formerly assistant to Professor Chiara, Prague, has been nominated to the directorship of the newly-founded School of Tropical Medicine in Western Australia. Dr. Breinl joined the Liverpool School of Tropical Medicine in 1904, and was appointed shortly afterwards to proceed to Manaos on the School's Yellow Fever Expedition.

It has been decided to proceed with the building of an extension to the Acton Cottage Hospital. The designs provide for a new children's ward, new operating room, new out-patients' ward, and committee room, and a new wing for accommodating the nurses. Mr. Alfred and Mr. Leopold Rothschild have given £400 towards the cost of the scheme, and £100 has been given anonymously.

Dr. H. D. Rolleston is Orator for the day at the inauguration of the new Session of St. George's Hospital Medical School, on October 1, 1909, at 3 o'clock, and will take for his subject: "St. George's and the Progress of Physic." The Oration will be followed by the annual meeting of the St. George's Club. The annual dinner will be held at the Prince's Restaurant in the evening, at 6.30 for 7 p.m. A. Marmaduke Sheild, Esq., F.R.C.S., Consulting Surgeon to the Hospital, in the chair. Tickets may be obtained from the Honorary Secretaries, Medical School Dinner, St. George's Hospital, S.W.

The number of deaths from pulmonary tuberculosis which occurred in Paris last year was 10,263. These figures are the same as for 1907, and as the population has slightly increased it may be concluded that consumption is decreasing in Paris.

The Duke of Devonshire will preside at the 21st annual Poor-law Conference for the counties of Derby, Leicester, Lincoln, Nottingham, and Rutland, at the Town Hall, Buxton, on October 21 and 22. Papers will be read on the Report of the Royal Commission on the Poor Laws in relation to rural unions, the proposed abolition of boards of guardians, and district nursing of the aged and destitute.

An appeal, signed by Mr. G. Marchetti, has been circulated among the Greek colony in London for funds in aid of the sufferers from the recent earthquake in Greece, by which two villages were entirely destroyed and hundreds of families rendered homeless. It is stated that the donations of persons not of Greek nationality will be welcomed. Cheques should be made to the order of, and forwarded to, Mr. George Marchetti, 57 Palmerston House, E.C. Contributions have already been received to the amount of over £600, including £40 from the Greek Minister.

It is reported that the greater part of the fortune of the late Mr. N. M. Wadia, C.I.E., of Bombay, which is stated to amount in the whole to £1,166,666, is to be administered by a trust for the relief of distress caused by fire and flood, earthquake and famine in any part of the world. Provision is also made for the establishment of hospitals, assisting medical institutions, defraying the expenses of marriages of poor girls of any nationality, providing for and educating orphans, assisting the destitute, and preserving animals useful to mankind.

Professor Osler, F.R.S., last week gave two lectures to the University Extension students assembled at Oxford, the first of which was on "The Beginnings of Modern Medicine." After showing the debt of medicine to Ancient Greece, he traced its growth through the Renaissance to the time when Harvey's little book, "De Motu Cordis," marked the final break of the modern spirit with the old traditions. The subject of the second lecture was "Michael Servetus." Professor Osler said he had not a shadow of doubt as to the full realisation by Servetus of the function of respiration on the blood. There had been an attempt made to deprive Servetus of the credit of the description, but it was a great deal better than those of others who claimed to be the first discoverers of the lesser circulation. The discovery of Servetus was his merit as a physiologist and it was one of the highest importance.

The fifth report of the Royal Patriotic Fund Corporation to the King for 1908 was published this week as a Blue Book [Cd. 4806].

Mr. Walter Malden, M.A., M.D., Cantab., M.R.C.S., St. Bartholomew's Hospital, has been admitted a member of the Royal College of Physicians.

Dr. Douglas Argyll Robertson, of Mon Plaisir, St. Aubins, Jersey, Hon. Surgeon Oculist to his Majesty the King in Scotland, consulting ophthalmic surgeon Edinburgh Royal Infirmary, left estate valued at £30,002. Dr. Rayner Winterbotham Batten, M.D., J.P., of 1 Brunswick Square, Gloucester, Senior Physician of Gloucester, a member of the Council of the British Medical Association, left estate valued at £46,973 gross, with net personalty £46,045.

According to the New York *Medical Record*, the physical examinations for marriage licences, as required by the new law in the State of Washington, is receiving the hearty support of the medical profession of that State. Their belief in the benefits to be derived from such a law, properly enforced, is shown by the resolution recently passed by the members of the Spokane County Medical Society agreeing to accept no fees for such examinations.

The annual report of the Registrar-General for Ireland, which has just been issued, states that during last year the population increased by 25,148, but against this increase there was a loss of 23,295 persons by emigration. The emigration was, however, more than 15,000 below the average for the past ten years. The total estimated population is now 4,371,455. The death-rate for the year was 17.6 per 1,000 or 0.1 below that for 1907 and 0.2 below the average rate for the preceding ten years. The highest death-rate was in the county borough of Dublin—24.0 per 1,000—whilst the lowest was in county Mayo—13.4 per 1,000.

We regret to announce the death of Professor Alphons von Rosthorn, the well-known chief of the Second Gynæcological Clinic of Vienna. Professor von Rosthorn had been ailing for some months, and was suffering from a cardiac affection, but his death last week came unexpectedly while he was absent on his annual holiday at his estate in Styria. As a gynæcologist he was universally esteemed, and during his successive terms of office as chief of the clinics at Prague, Heidelberg, and finally at Vienna he gained the respect and admiration of the many English and American graduates who flocked to work under him. All who came into contact with him carried away the pleasantest of recollections of the chief, whose death, at a comparatively early age and while in the prime of his career, comes as a personal loss to everyone who has had the privilege of working under him. Professor von Rosthorn was a fine diagnostician and operator, and contributed largely to the literature of his speciality. He was also interested in hospital construction, and much of the excellence of the new Gynæcological Clinic at Vienna is due to the personal supervision that he exercised over the plans and to the improvements he suggested.

